# Low concentration cell painting images enable the identification of highly potent compounds

**DOI:** 10.1038/s41598-024-75401-5

**Published:** 2024-10-17

**Authors:** Son V. Ha, Steffen Jaensch, Lorena G. A. Freitas, Dorota Herman, Paul Czodrowski, Hugo Ceulemans

**Affiliations:** 1 Janssen Pharmaceutica, N.V., a Johnson & Johnson company, 2340 Beerse, Belgium; 2 Department of Chemistry, Johannes Gutenberg University Mainz, Mainz, Germany

**Keywords:** Biochemistry, Cell biology, Chemical biology, Computational biology and bioinformatics, Drug discovery

## Abstract

Image-based models that use features extracted from cell microscopy images can estimate the activity of small molecules in various biological assays. Typically, models are trained on images stained by an optimized protocol (e.g. Cell Painting) after exposure to a fairly high small molecule concentration (referred to as ’image concentration’) of $10\; \upmu {\text{M}}$ or higher. Low concentration images (e.g. $0.16$ μM, $0.8$ μM, $4$ μM) tend to yield models with worse performance. In this work, we nevertheless report a practical use for low image concentration data. We propose the combination of well-performing models trained at higher image concentrations, with lower image concentration for inference to identify more potent compounds. We show that this approach improves on the conventional method (directly training a high-potency model) in 65$\%$ of assays investigated in terms of AUC-ROC, and 75$\%$ of assays in terms of RIPtoP-corrected AUC-PR.

## Introduction

High-throughput screening has been a powerful tool to identify hits for a primary assay from large libraries of small molecules. Hits are then grouped by structure similarity into chemical series, which act as a starting point for further prioritization and optimization. Series are prioritized and optimized based on not only their primary activity, but also on their secondary activities - physicochemical, pharmacokinetic, and toxicological properties^1^. Here, quantitative structure-activity relationship (QSAR) models are often used to optimize primary and secondary activities, by modelling activities using chemical descriptors such as ECFP^2^ or SMILES^3^.

**Fig. 1:**
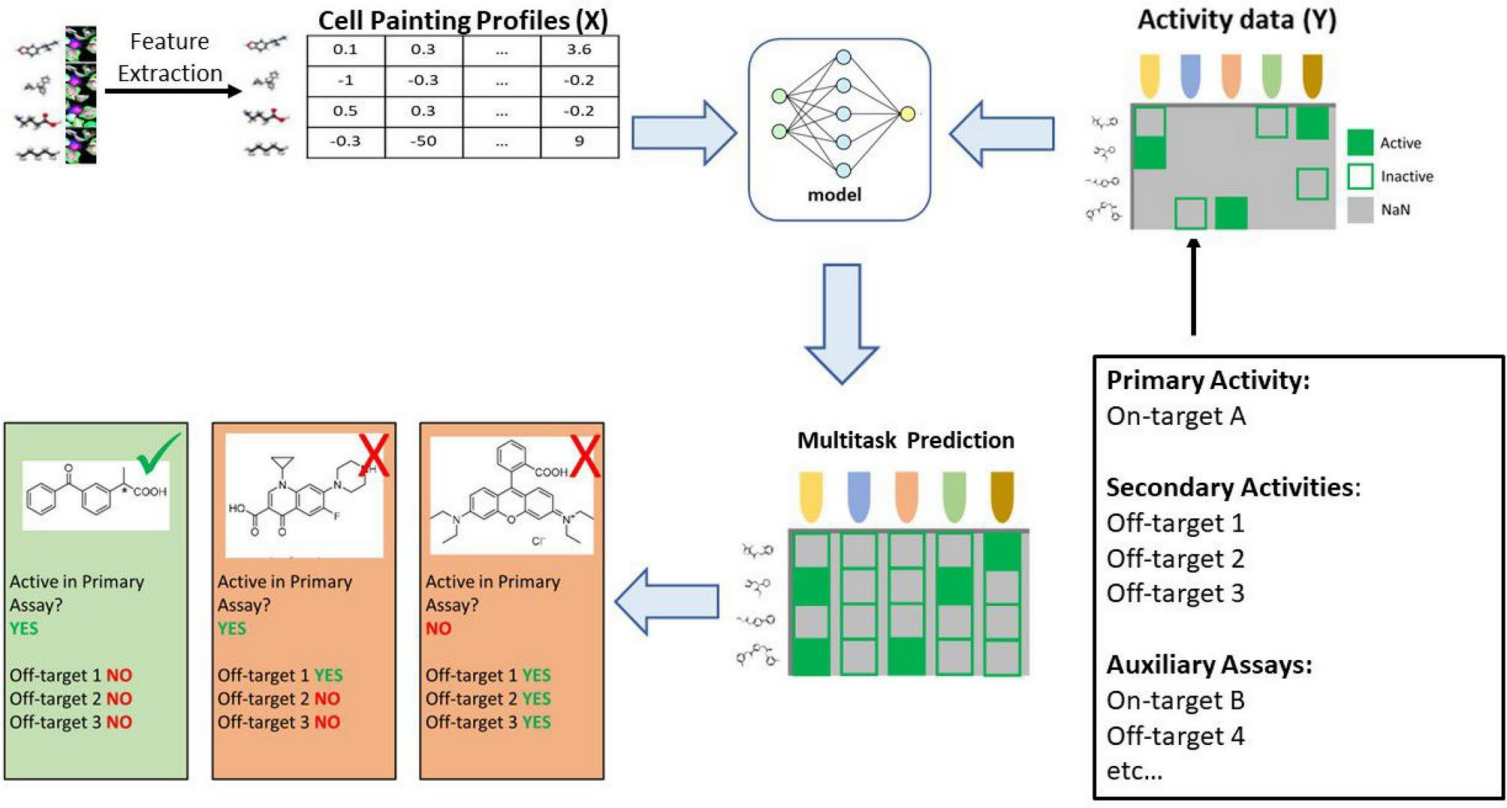
Image-based Multitask Activity Model. Using features extracted from Cell Painting images as input, and a sparse activity matrix as labels, the model learns to fill in the activity values for other compounds. These estimated activity values can be used for prioritizing and further optimizing chemical series.

Image-informed ligand-based models (Fig. 1) or image-based models for short, are another paradigm for activity optimization, where instead of chemical descriptors, features extracted from cell microscopy images are used as input. These images capture the morphological changes of cells induced by chemical compounds in a standardized protocol. Image-based models have been shown to increase assay hit rates and chemical hit diversity over their initial high throughput screening campaigns^4^, estimate mitochondrial toxicity^5^, and expand the applicability domain of QSAR models^1^. Currently, the most popular staining protocol for this application is Cell Painting^6^.

**Fig. 2:**
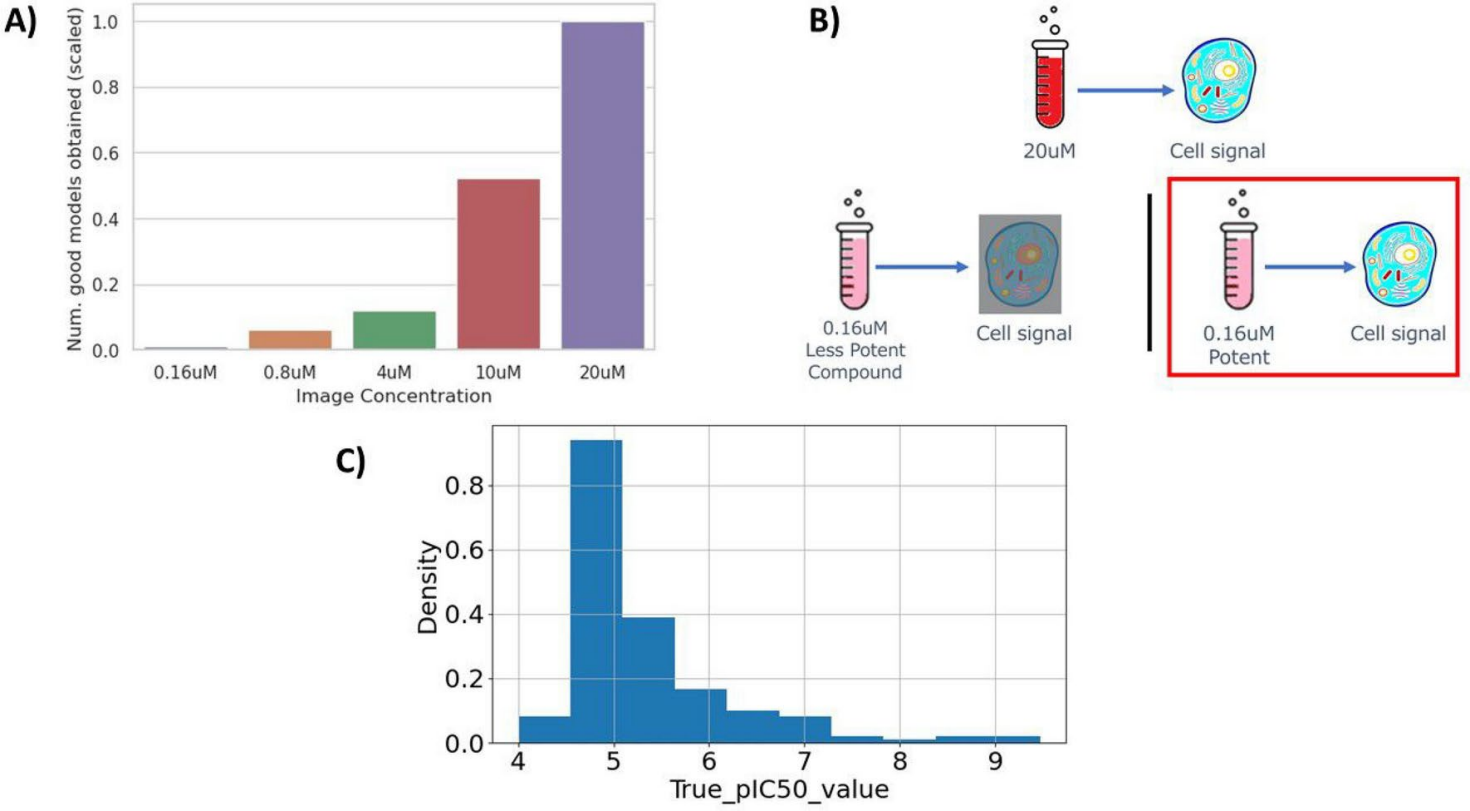
(**A**) Number of assays for which we could build classification models with ROC-AUC$\ge 0.8$. Results from a previous internal study. As image concentration increases, the more models with ROC-AUC$\ge 0.8$ we obtain. Bar height are scaled by a factor equal to the original height of column ‘$20\; \upmu {\text{M}}$’. This result was obtained by training the same multitask bioactivity model end-to-end with the identical training procedure for each image concentration. We also used the same evaluation procedure to calculate the ROC-AUC score for each task, subsequently quantifying the number of tasks per image concentration that reached a ROC-AUC$\ge 0.8$. Details of the model, training procedure and evaluation procedure as can be found in Herman et al., under Experimental Procedures/Image-Informed Ligand-Based Model Building and Experimental Procedures/Model Evaluation. (**B**) Intuition of the behavior of low concentration images. High concentration images show a lot more cell signals than low concentration images, which makes them more suitable for modelling. In low concentration images, only highly potent compounds induce signal from cell. (**C**) Distribution of true pIC50 values of ‘assay 24’ in the training set. We consider compounds with pIC50$\ge$5 to be moderately potent, and pIC50$\ge$7 to be highly potent. Training a classifier for the latter tend to be more difficult than the former, due to data imbalance (few positive samples).

Much of the research on Cell Painting images are based on two public datasets: 30K compounds from Bray et al^6^ and JUMP-CP^7^, both primarily contain images acquired at $10\; \upmu {\text{M}}$ concentration of test compound. Here we study an internal dataset acquired at five image concentrations $0.16\; \upmu {\text{M}}$, $0.8\; \upmu {\text{M}}$, $4\; \upmu {\text{M}}$, $10\; \upmu {\text{M}}$ and $20\; \upmu {\text{M}}$. For activity modelling, according to a previous internal study, we found that the higher the image concentration, the higher the ratio of classification models with a ROC-AUC$\ge 0.8$ we obtained (Fig. 2A). This result encourages using high concentration images such as $10\; \upmu {\text{M}}$ and $20\; \upmu {\text{M}}$ to train activity models.

Why do lower image concentrations result in poorer model performance than high image concentrations? Signal amplitude in the image features tends to increase with compound concentration (Fig. 2B), and that a minimal signal amplitude is required for modelling. Less potent compounds, which are more abundant in chemical libraries, will require higher concentrations to induce model-compatible signal than more potent compounds, which are rarer. We propose that once a well performing model has been trained and validated at higher image concentrations, where enough compounds induce signal to enable modelling, it can be repurposed without retraining to detect similar but rarer signal at lower image concentrations, implying higher potency.

One benefit of repurposing a moderate-potency model for highly potent compound retrieval is overcoming data imbalance. Typically, when building a highly potent compound classifier, one very common problem is the shortage of positive samples (Fig. 2C) affecting model training. As a result, we generally obtain much fewer good high-potency models than moderate-potency models. Our approach can facilitate highly potent compound retrieval in assays which only have enough data to train a good moderate-potency model.

In this work, we investigate the behavior of models trained on $20$ μM images and inference performed with low concentration images $0.16$ μM, $0.8$ μM and $4$ μM. We show this approach can be used across a broad range of assays to repurpose a moderate-potency model for highly potent compound retrieval with high accuracy. In a drug discovery campaign, this can help prioritize more potent hits from virtual screening for experimental follow-up, and deprioritize compounds with potent off-target activities in the hit-triaging phase. We also compare our approach to the conventional method (directly building a high-potency classifier) in correctly classifying highly potent compounds. We show that this holds true for 65$\%$ of the assays in terms of AUC-ROC, and 75$\%$ of assays in terms of RIPtoP corrected AUC-PR.

## Methods

### Data and model description

#### Cell painting image acquisition and processing

 Images of chemically perturbed cells were acquired using Cell Painting, a high-content image-based assay for morphological profiling. In short, U2OS cells purchased from ATCC (HTB-96) were seeded in 1536 well plates and allowed to attach for 24 h. Compounds were diluted in DMSO to the final concentration, either $20\; \upmu {\text{M}}$, $10\; \upmu {\text{M}}$, $4\; \upmu {\text{M}}$, $0.8\; \upmu {\text{M}}$ or $0.16\; \upmu {\text{M}}$. We label different cell components or organelles using the same fluorescent dyes as described in Bray et al^6^. Images of the five fluorescence channels were acquired with a Yokogawa (Tokyo, Japan) CellVoyager 8000 confocal high-content imaging reader. Then the PerkinElmer (Waltham, MA) Acapella version 4.1.3 (https://content.perkinelmer.com/lab-products-and-services/product-support.html) image analysis software was used to extract around 800 morphological features from individual cells, such as staining intensity, texture, shape and spatial correlations. After that, quality control, well-level aggregation and normalization against the DMSO control on every plate are performed. In the end, we obtain a vector of Z-scores for each compound, which we will call a Cell Painting profile. More details about the Cell Painting protocol and image processing can be found in Herman et al^1^ under Experimental Procedure/Data Preparation/Cell Painting Images and Image Preprocessing.

#### Multitask image-based activity modelling

 We form activity modelling as a multitask learning (MTL) problem, where a single model is trained on multiple tasks (binarized assays) simultaneously. The aim of MTL is to improve modelling capability of each task by leveraging the correlation of multiple tasks modelled jointly.

All modelling tasks are binary classification tasks, obtained from binarizing bioactivity assays which originally measures potency of a compound. Potency is measured as IC50 and correspond to the concentration at which a compound elicits 50$\%$ of a desired effect (e.g. inhibition). In line with previous literature, we use the pIC50 which is the IC50 on logarithmic scale, calculated as -log10(IC50).

In our case, we have over a hundred thousand Cell Painting profiles, and thousands of assays to train and evaluate the model. We set aside $<5\%$ of the Cell Painting profiles for evaluation, and the rest is used for training. Compounds whose Cell Painting profiles are in the test set satisfy two criteria: (1) Have pIC50$\ge$8 in at least one of the bioassays, (2) Have a unique Murcko Scaffold^8^ compared to the training set. The rest of the Cell Painting profiles are used for model training. Since the fill rate of the matrix is very low (around 5$\%$), we use a masked Binary Cross-Entropy (BCE) loss where if we don’t have activity information about a compound for a specific task, we exclude that (compound, task) pair from loss calculation.

#### Training set folds split

 Cell Painting profiles in the training set are split into 5 folds, based on the Murcko Scaffolds of the original compound. We ensure that each fold only contains Cell Painting profiles of compounds which are structurally closest to each other. These folds will be used for Mondrian Cross-Conformal Prediction, as described below.

#### Model

 We use an ensemble of 8 Multi-Layer Perceptrons (MLPs), calibrated using Mondrian Cross-Conformal Predictor (MCCP)^9^. Details of the ensemble and each MLP architecture can be found in the Supplementary Information, along with the hyperparameters for each model.

MCCP is a conformal prediction method designed to define prediction confidence in large imbalanced dataset, such as bioactivities. Our implementation of MCCP closely follows that in Sun et al.^9^. Given a calibration set, we define the **conformity measure** (CM) of a probability score output as $Conformity Measure (label, output) = |1-label-output|$, where $label=1$ indicates active, $label=0$ indicates inactive, and *output* is the probability score output from the model. Let the CM of the outputs in the calibration set for active class be $\alpha _1$, $\alpha _2$ ... $\alpha _n$, and for inactive class be $\beta _1$, $\beta _2$ ... $\beta _m$. If the CM for a new output is $\alpha _t$ (for label active) and $\beta _t$ (for label inactive),then p-values for active is $p^t_1 = \frac{|{i = 1,..,n: \alpha _i\le \alpha _t|}}{n}$, and p-values for inactive is $p^t_0 = \frac{|{i = 1,..,m: \beta _i\le \beta _t|}}{m}$.

In addition, a process similar to k-fold cross-validation is used. The training set is divided into k equal folds, one fold will be chosen as a calibration set for p-value calculation, and the other k-1 folds are used to train the model. The process is repeated k times, with each fold being used as the calibration set exactly once. The p-values from these repeats are then averaged to produce a single inactive p-value $p^t_0$ and active p-value $p^t_1$ for the new output.

Using significance $\varepsilon$, we obtain the conformal score for the new output as follows:

Active: $$p^t_1 > \varepsilon$$ and $$p^t_0 \le \varepsilon$$

Inactive: $$p^t_0 > \varepsilon$$ and $$p^t_1 \le \varepsilon$$

Uncertain: ($p^t_0 > \varepsilon$ and $p^t_1 > \varepsilon$) or ($p^t_0 \le \varepsilon$ and $p^t_1 \le \varepsilon$)

#### Training and inference process

 A visualization of the process can be found in the Supplementary Information. Given the test set and the training set which has been split into 5 folds, model training and inference process is as follows:

1. Train the model, which is an ensemble of 8 MLPs, on the entire training set so that we can get the best model possible. We minimize masked BCE loss using Adam optimizer^10^, and train the model for 34000 iterations. After that, compute probability scores for the test set.
2. Perform MCCP for the 5 folds of the training set. For each calibration fold choice, train the model on the other 4 folds. Calculate active and inactive p-values for each calibration fold, then average the p-values to obtain a single active p-value and inactive p-value that represents the overall level of significance of the probability scores.
3. Obtain the conformal scores for test set at significance $\varepsilon = 0.05$. The pipeline will return 1 for Active, -1 for Inactive, and 0 for Uncertain.

### Usage of low concentration images for highly potent compounds retrieval

Our approach involves modifications to the above training and inference process: Using high concentration images for training and low concentration images for inference. This step repurposes a good moderate-potency model for highly potent compound retrieval. For example, if there is an assay with enough positive and negative samples to build a good model at potency threshold pIC50 $\ge$ 5, and the aim is to retrieve highly potent compounds with pIC50 $\ge$ 7, our approach is as follows:

Step 1: Train a model using $20\; \upmu {\text{M}}$ images to classify active compounds at potency threshold pIC50 $\ge$5.    With abundant phenotypes from high concentration cell images, the model can learn to recognize signal specific to different bioassays. Besides, at this potency threshold 5, there are plenty of labelled data such that data imbalance is not an issue.

Step 2: Inference using low concentration input images As aforementioned, only highly potent compounds induce signals from cells at low compound concentration. Hence, if we switched to low concentration input images for inference, only highly potent compounds are identified as active.As a result, the model originally trained to classify compounds at pIC50$\ge$5 can be used to classify compounds at a higher potency threshold (e.g. pIC50$\ge 7$).

## Results

For conciseness, we name our model using this convention: [Train image concentration/Inference image concentration/train pIC50 label threshold]. For example, a model which uses $20\; \upmu {\text{M}}$ images for training, $0.16\; \upmu {\text{M}}$ images for inference, and learns to classify compounds with pIC50$\ge$ 5 will be named as a $[20\; \upmu {\text{M}} / 0.16\; \upmu {\text{M}} / 5]$ model.

We report results on 57 assays. The criteria for choosing these 57 assays are:

- In the train set, each task from each assay has at least 100 labelled datapoints, and among those there are at least 25 actives and 25 inactives (at both potency threshold 5 and 7).
- In the test set, each task from each assay has at least 10 total labelled datapoints, and among those there are at least 5 actives and 5 inactives (at both potency threshold 5 and 7).
- We can obtain a good $[20\; \upmu {\text{M}} / 20\; \upmu {\text{M}} / 5]$ for this assay. (Criterion: AUC-ROC $\ge$ 0.7 in a cross-validation process similar to Herman et al.^1^.)

### Results from one assay

**Fig. 3:**
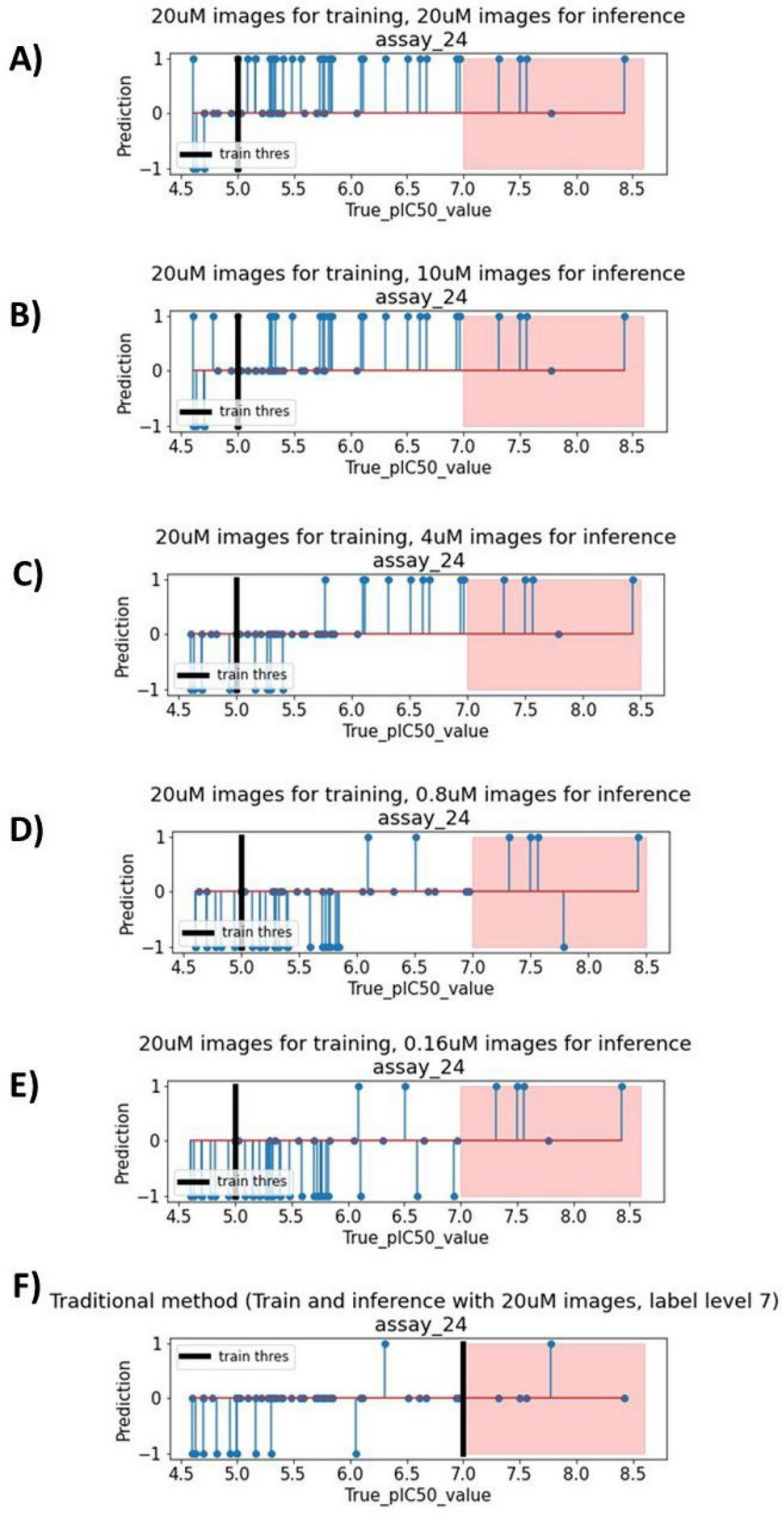
Stem plots showing model conformal scores against the true pIC50 value. For each plot, the y-axis denotes the conformal score for each compound. A score of 1 is positive, -1 is negative, and 0 is out of domain. The vertical black line denotes the potency threshold of the label the model was trained on. The red area denotes the range of pIC50 which we consider highly potent (in this case pIC50$\ge$7). The model behaves as a normal pIC50$\ge$5 classifier in plot A). But when using low concentration images for inference, the model specifically retrieves highly potent compounds in the red region, and skips over the moderately potent compounds. This behavior is particularly clear in plot D) and E). In fact, these two plots show, out of five highly potent compounds, our approach retrieve fours, whereas the conventional method in plot F) can only retrieve one.

Firstly, we give a detailed visualization of the results from one randomly chosen assay (named ‘assay_24’). Our aim is to intuitively visualize the behavior of the model when using low concentration images for inference.

All stem plots in Fig. 3 show the outputs of the models on the y-axis, and the true experimental pIC50 value on the x-axis. The black line denotes at what threshold pIC50 labels are binarized at, and the red area denotes the range of pIC50 we consider highly potent. For example, Fig. 3A shows the result of a standard $[20\; \upmu {\text{M}} / 20\; \upmu {\text{M}} / 5]$ model. In this situation, the model behaves as expected: Most compounds to the right of the black line (True_pIC50_value$\ge$ 5) are classified as 1 (active), and compounds to the left of the black line are mainly classified as -1 (inactive) or 0 (uncertain) by the model. This is clearly a pIC50$\ge$ 5 model and using it for classifying pIC50$\ge$ 7 will result in many false positives. Figure 3C–E show what happens when we use the same $20\; \upmu {\text{M}}$ pIC50$\ge$ 5 model, but perform inference with features from lower concentration images instead (still of the same compounds). It can be observed that the model now classifies less compounds as active, and tends to specifically retrieve compounds in the highly potent range (red area), skipping almost all of the moderately potent compounds. In fact, both models [$20\; \upmu {\text{M}}$/$0.16\; \upmu {\text{M}}$/5] and [$20\; \upmu {\text{M}}$/$0.8\; \upmu {\text{M}}$/5] classify highly potent compounds with high precision (4 correct positive classifications out of 6 positive classifications made by the model).

Figure 3F shows the result of a high-potency $[20\; \upmu {\text{M}} / 20\; \upmu {\text{M}} / 7]$ model, dubbed as the ‘conventional method’ since this is what is typically done for retrieving compounds with pIC50$\ge$ 7. This conventional method employs the same training and inference process outlined in the Methods section, excluding the modification described in the “Usage of Low Concentration Images for Highly Potent Compounds Retrieval” subsection. We believe this is a suitable representation of current methods as it mirrors previous works^1,4,11,12^, that performed multitask bioactivity modeling using only one image concentration.

In this case, due to data imbalance affecting high-potency model (assay pIC50 distribution in Fig. 2C), the model severely underperforms. It manages to only retrieve one highly potent compound (pIC50$\ge$ 7) out of five. In contrast, models using low concentration images for inference $[20\; \upmu {\text{M}} / 0.8\; \upmu {\text{M}} / 5]$ and $[20\; \upmu {\text{M}} / 0.16\; \upmu {\text{M}} / 5]$ can both retrieve four out of five highly potent compounds from this assay.

Based on the above results, we hypothesize two points regarding the use of low concentration images in retrieving highly potent compounds:

- **Hypothesis 1**: A good moderate-potency model e.g. $[20\; \upmu {\text{M}} / 20\; \upmu {\text{M}} / 5]$ can be repurposed to specifically retrieve compounds with higher potency (e.g pIC50$\ge$ 7) simply by switching to features from low concentration images for inference (without retraining the model).
- **Hypothesis 2**: In case of data imbalance adversely affecting training of a high-potency model, our approach can improve upon the high-potency model (‘conventional method’) in retrieving highly potent compounds.

We will further investigate these two points when showing results from other assays in the next sections.

### Results from 57 assays

**Table 1:** Assay type for 57 assays.

Assay type	Assay index
Other targets	0, 3, 4
Kinase	1, 2, 6–13, 56
Cell proliferation	5, 14–55

**Fig. 4:**
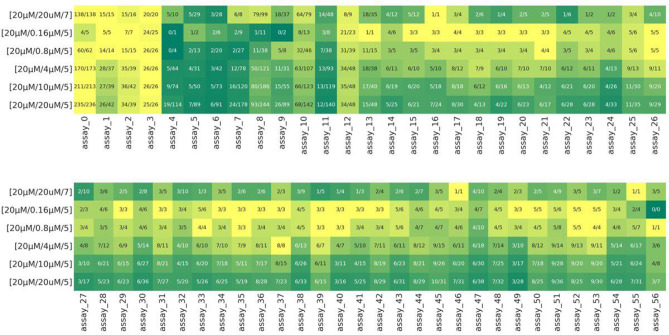
High-potency precision heatmap. Recorded high-potency precision of each model across 57 assays. As the inference image concentration decreases (moving from the sixth row to the second row), the model tends to be more precise at classifying highly potent compounds, resulting in a lighter color. This color gradient is consistent across all assays. The top rows are precision scores of the conventional method.

**Fig. 5:**
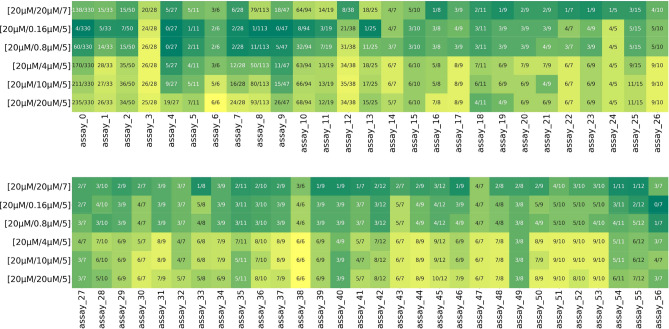
High-potency recall heatmap. Recorded high-potency recall of each model across 57 assays. As the inference image concentration decreases (moving from the sixth row to the second row), the model’s recall decreases, resulting in a darker color. This color gradient is consistent across all assays. The top row are recall scores of the conventional method.

**Figure 6:**
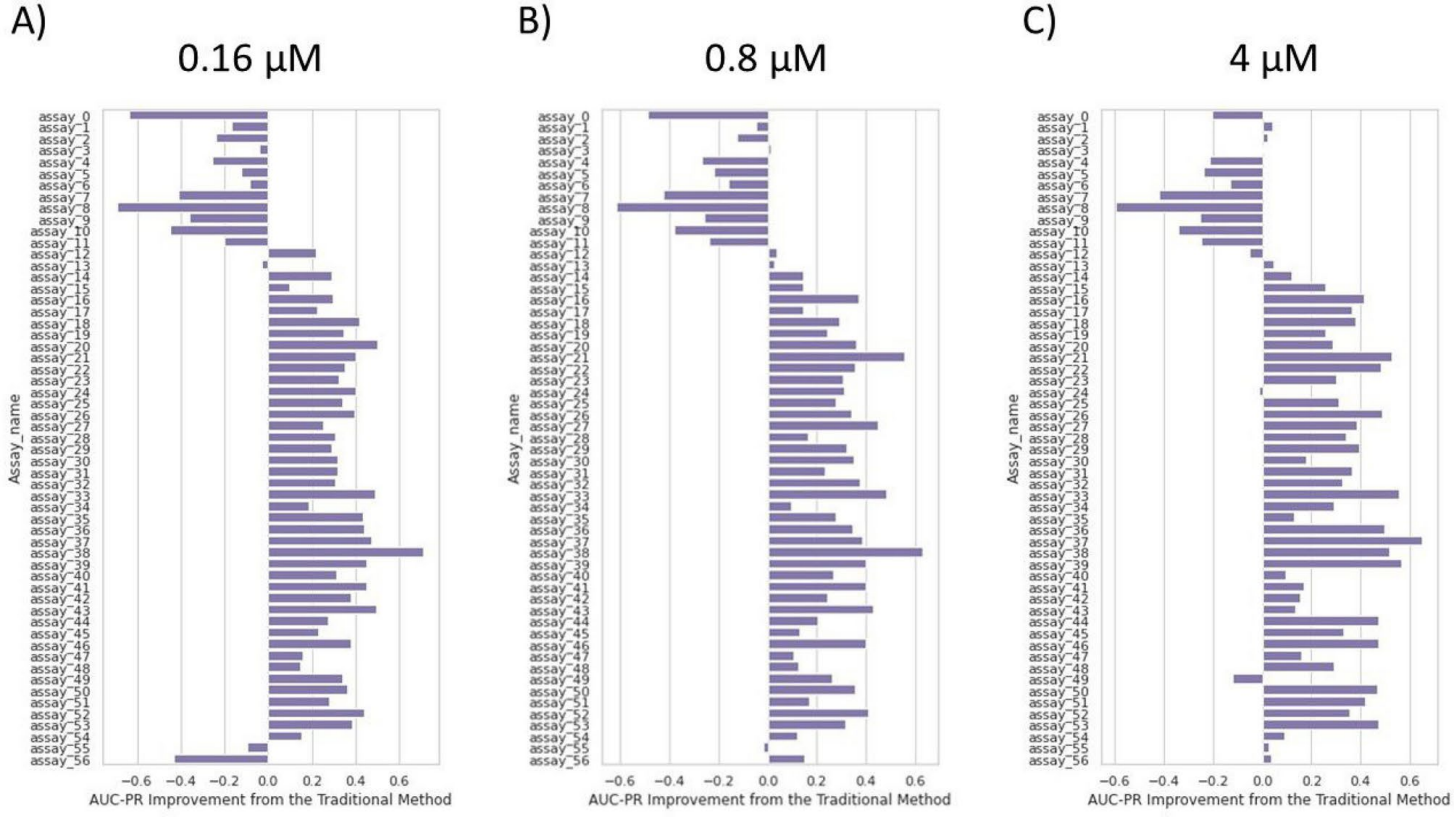
AUC-PR improvement when using our approach compared to the conventional method. Results across 57 assays. Our approach improves AUC-PR in 75$\%$ of assays investigated, with improvements around 0.2 to 0.5 compared to the conventional method.

**Figure 7:**
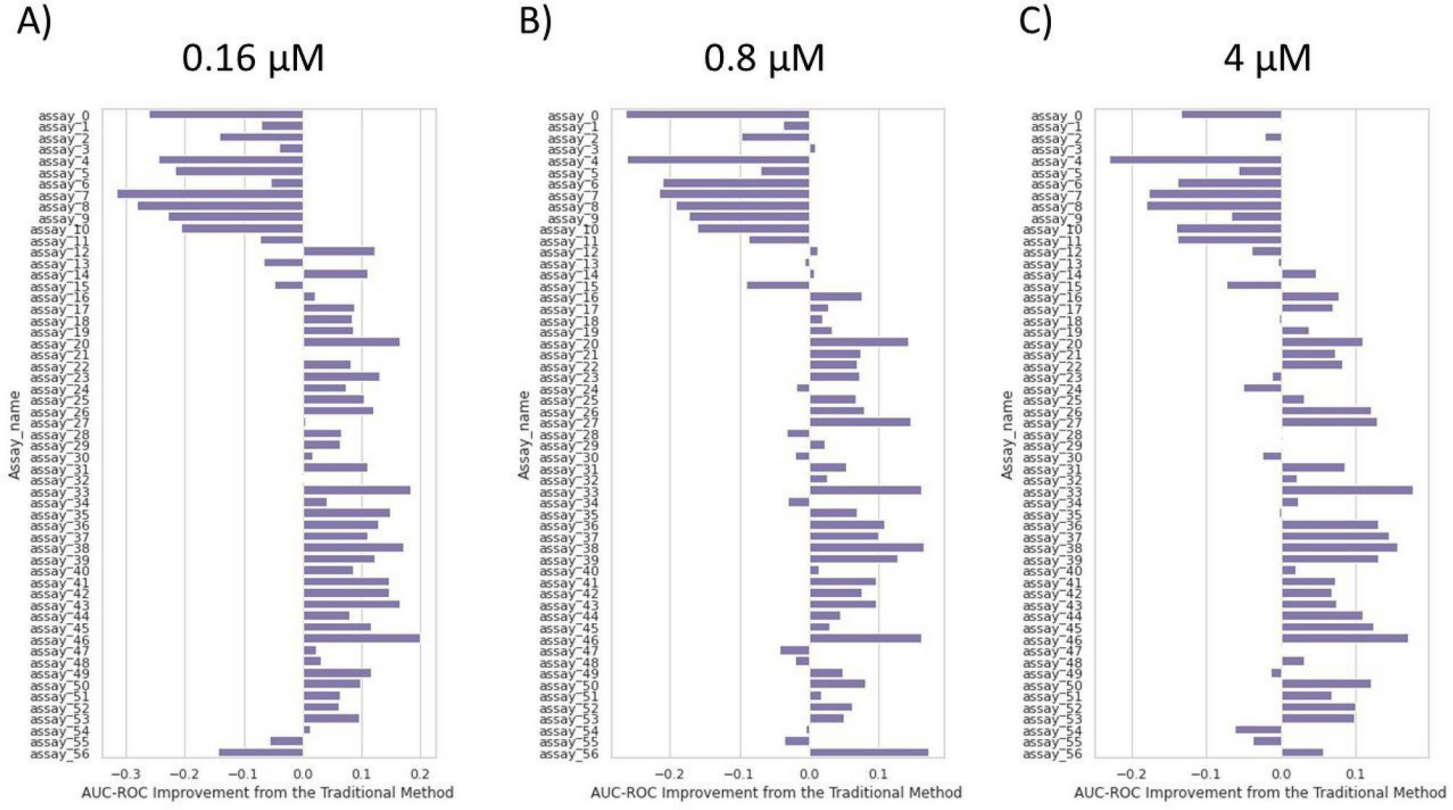
AUC-ROC improvement when using our approach compared to the conventional method. Results across 57 assays. Our approach improves AUC-ROC in 65$\%$ of assays investigated, with improvements around 0.1 to 0.2 compared to the conventional method.

To assess **Hypothesis 1**, we calculate the precision in classifying highly potent compounds pIC50 $\ge$ 7 (high-potency precision). This is because if e.g. the $[20\; \upmu {\text{M}} / 0.16\; \upmu {\text{M}} / 5]$ model is specifically retrieving highly potent compounds, then out of the compounds classified active by the model, the majority of them should be highly potent. In contrast, the $[20\; \upmu {\text{M}} / 20\; \upmu {\text{M}} / 5]$ model would have very low high-potency precision.

High-potency precision is calculated and plotted on a heatmap in Fig. 4 for all 57 assays and displayed in the form of a fraction: $\#$True Positives/$\#$(True Positive + False Positive). For most assays, using low concentration images ($0.16\; \upmu {\text{M}}$, $0.8\; \upmu {\text{M}}$, $4\; \upmu {\text{M}}$) for inference increases high-potency precision compared to the $[20\; \upmu {\text{M}} / 20\; \upmu {\text{M}} / 5]$ model. For models $[20\; \upmu {\text{M}} / 0.16\; \upmu {\text{M}} / 5]$ and $[20\; \upmu {\text{M}} / 0.8\; \upmu {\text{M}} / 5]$, high-potency precision in many assays is between 0.8 to 1. In addition, when using low concentration images for inference, fewer compounds are classified as active, but these compounds are very likely to be highly potent. It is also worth pointing out that high-potency precision of the models $[20\; \upmu {\text{M}} / 0.16\; \upmu {\text{M}} / 5]$ and $[20\; \upmu {\text{M}} / 0.8\; \upmu {\text{M}} / 5]$ is higher compared to the conventional method $[20\; \upmu {\text{M}} / 20\; \upmu {\text{M}} / 7]$ (Fig. 4 top row) in almost all 57 assays.

However, the improvement in high potency precision comes with decreasing recall (Fig. 5) when using high concentration images for inference. Though notably among the low concentration images, $4\; \upmu {\text{M}}$ images achieve significantly higher recall than $0.8\; \upmu {\text{M}}$ and $0.16\; \upmu {\text{M}}$ images while demonstrating recall comparable to, or only slightly worse than $10\; \upmu {\text{M}}$ and $20\; \upmu {\text{M}}$ images when used for inference with the moderate-potency models. In addition, high-potency recall of the conventional method (e 5 top row) is lower than those of model $[20\; \upmu {\text{M}} / 4\; \upmu {\text{M}} / 5]$ for most of the 57 assays.

Overall, regarding hypothesis 1, we observe that using low concentration images for inference substantially improves high potency precision at the expense of recall compared to high concentration images. This pattern is consistent across all 57 assays investigated. In drug discovery practice, depending on the use case, higher precision may be more beneficial than higher recall. For example when using such models for an image-based virtual screen of a large compound library with a low throughput follow-up assay, or during hit triaging to deprioritize only compounds with potent off-target activities while minimizing the risk of false positive off-target flagging.

To assess **Hypothesis 2**, we propose to use Area under the ROC Curve (AUC-ROC) and Precision-Recall curve (AUC-PR). These are common metrics to compare classification models. we apply Relative Improvement of Proximity to Perfection (RIPtoP) correction to AUC-PR, as in Heyndrickx et al^13^. The idea of RIPtoP correction is that: since the random baseline of AUC-PR for each assay is the active ratio, one cannot compare AUC-PR across 2 different assays. RIPtoP correction enables cross-assay AUC-PR comparison by effectively ‘rescaling’ AUC-PR so that for every assay, 1 is the perfect model and 0 is the random baseline. The formula for RIPtoP correction is:

$$RIPtoP(AUC\text {-}PR) = \frac{AUC\text {-}PR \ - \ BASELINE}{1 \ - \ BASELINE}$$

Figure 6 shows how much AUC-PR improves over the conventional method when using for inference A) $0.16\; \upmu {\text{M}}$, B) $0.8\; \upmu {\text{M}}$ and C) $4\; \upmu {\text{M}}$ images. Out of 57 assays, our approach improves AUC-PR by 0.2 to 0.5 in 42, 45 and 45 assays, respectively, over the conventional method, which equates to an improvement in AUC-PR in roughly 75$\%$ of assays investigated. Similarly, Fig. 7 shows out of 57 assays, using A) $0.16\; \upmu {\text{M}}$, B) $0.8\; \upmu {\text{M}}$ and C) $4\; \upmu {\text{M}}$ images for inference improves AUC-ROC in 40, 36 and 34 assays, respectively, over the conventional method. This equates to an improvement in AUC-ROC in approximately 65$\%$ of assays investigated, with AUC-ROC improvements generally around 0.1 to 0.2. Examining the assay types of the 57 assays in Table 1 reveals that the subset where our approach performs relatively poorly (assay 0 to assay 13, and assay 56) primarily consists of kinase and other target assays. In contrast, our approach significantly improves AUC-ROC and AUC-PR for cell proliferation assays.

Overall, hypothesis 2 holds for most but not all assays investigated (approximately 65$\%$ or 75$\%$ based on AUC-ROC and AUC-PR, respectively). Additionally, we find that hypothesis 2 primarily holds for cell proliferation assays.

## Case studies

In the previous section, we report results for 57 assays chosen using a list of criteria listed at the beginning of the Result section. Since we need a systematic way to evaluate performance, the assay criteria are fairly rigid, hence there is low assay diversity. In fact, 43 out of 57 assays are Cell Proliferation assays. The others are 11 kinase assays, and 3 assays on other protein targets.

Therefore, in this section, to increase assay diversity, we manually pick a number of off- and on-target assays to test our low concentration image method on. These assays are different from the previous 57 assays in several ways: the pIC50 threshold where we consider a compound moderately or highly potent can be different, or there are plenty of positive samples, or the assays can even measure something that is not pIC50. Our aim is to demonstrate how we apply the method in these different settings, and in which setting the method works well.

### Phospholipidosis assay

**Table 2:** PLD metrics table. A high potency precision increase as inference image concentration decreases can be observed, indicating that the pIC50$\ge$4.6 model has been repurposed for retrieving highly potent compounds with pIC50$\ge$6. The best model in terms of high potency AUC-ROC and AUC-PR is $[20\; \upmu {\text{M}} / 4\; \upmu {\text{M}} / 4.6]$, outperforming the conventional method $[20\; \upmu {\text{M}} / 20\; \upmu {\text{M}} / 6]$.

Model name	High potency precision	High potency AUC-PR	High potency AUC-ROC
$[20\; \upmu {\text{M}} / 0.16\; \upmu {\text{M}} / 4.6]$	0 (0/0)	0	0.5
$[20\; \upmu {\text{M}} / 0.8\; \upmu {\text{M}} / 4.6]$	0.429 (3/7)	0.00512	0.513
$[20\; \upmu {\text{M}} / 4\; \upmu {\text{M}} / 4.6]$	0.286 (26/91)	**0.0970**	**0.731**
$[20\; \upmu {\text{M}} / 10\; \upmu {\text{M}} / 4.6]$	0.243 (27/111)	0.0689	0.693
$[20\; \upmu {\text{M}} / 20\; \upmu {\text{M}} / 4.6]$	0.209 (27/129)	0.0414	0.636
$[20\; \upmu {\text{M}} / 20\; \upmu {\text{M}} / 6]$	**0.462 (6/13)**	0.0332	0.556

Highest values are in bold.

Drug-induced phospholipidosis (PLD) is characterized by the excess accumulation of phospholipids in tissues. Organs affected by phospholipidosis exhibit inflammatory reactions and histopathological changes^14^. Hence, PLD is considered an adverse effect and PLD assay is an essential liability assay to screen in early drug development.

For PLD assay, the model with the best AUC-ROC we have is the pIC50$\ge$4.6 model, which is the lowest potency threshold considered for this assay. The model with the highest potency threshold is pIC50$\ge$6, which has the worst AUC-ROC out of all our PLD models. The active ratio in the training set, when potency threshold is at 4.6 and 6, is 0.776 and 0.109, respectively. Our aim is to investigate whether we can repurpose a pIC50$\ge$4.6 model to a model which specifically retrieves highly potent compounds pIC50$\ge$6 (**Hypothesis 1**), and whether this method can outperform the pIC50$\ge$6 model (**Hypothesis 2**).

It can be seen from the increase in high potency precision from 0.209 to 0.429 in Table 2 that using low concentration images for inference can help specifically retrieve highly potent compounds, indicating that Hypothesis 1 holds. Although for this case, it is interesting to note that the model with the highest high potency precision is $[20\; \upmu {\text{M}} / 20\; \upmu {\text{M}} / 6]$ at 0.462. The low concentration model $[20\; \upmu {\text{M}} / 8\; \upmu {\text{M}} / 4.6]$ comes close at 0.429.

Hypothesis 2 holds, as the best model in terms of high potency AUC-PR and AUC-ROC is $[20\; \upmu {\text{M}} / 4\; \upmu {\text{M}} / 4.6]$, achieveing 0.0970 and 0.731, respectively. On the other hand, model $[20\; \upmu {\text{M}} / 20\; \upmu {\text{M}} / 6]$ AUC-ROC is close to the random baseline 0.5, likely due to few positives to train the model at pIC50 threshold 6, as the active ratio at potency threshold 6 is only 0.109. Interestingly, model $[20\; \upmu {\text{M}} / 20\; \upmu {\text{M}} / 6]$ achieves the highest high potency precision, but relatively low high potency AUC-PR and AUC-ROC at the same time. This is because this model misclassifies a lot more positives compared to other models.

### BSEP assay

**Table 3:** BSEP metric table. A high potency precision increase as inference image concentration decreases can be observed, indicating that the pIC50$\ge$4.5 model has been repurposed for retrieving highly potent compounds with pIC50$\ge$5.5. The best model in terms of high potency AUC-ROC and AUC-PR is $[20\; \upmu {\text{M}} / 20\; \upmu {\text{M}} / 5.5]$. In this case, our method does not outperform the conventional method.

Model name	High potency precision	High potency AUC-PR	High potency AUC-ROC
$[20\; \upmu {\text{M}} / 0.16\; \upmu {\text{M}} / 4.5]$	**1.0 (1/1)**	0.0108	0.505
$[20\; \upmu {\text{M}} / 0.8\; \upmu {\text{M}} / 4.5]$	**1.0 (15/15)**	0.161	0.581
$[20\; \upmu {\text{M}} / 4\; \upmu {\text{M}} / 4.5]$	**1.0 (62/62)**	0.667	0.833
$[20\; \upmu {\text{M}} / 10\; \upmu {\text{M}} / 4.5]$	0.908 (69/76)	0.632	0.848
$[20\; \upmu {\text{M}} / 20\; \upmu {\text{M}} / 4.5]$	0.721 (75/104)	0.445	0.808
$[20\; \upmu {\text{M}} / 20\; \upmu {\text{M}} / 5.5]$	0.895 (77/86)	**0.689**	**0.885**

Highest values are in bold.

Bile salt export pump (BSEP) is the major transporter for the secretion of bile acids from hepatocytes into bile in humans. BSEP inhibition may contribute to the initiation of human drug-induced liver injury (DILI)^15^. Since DILI is a frequent cause of drug failure in development, early screening of BSEP is also vital in early drug discovery.

For BSEP assay, pIC50$\ge$4.5 and pIC50$\ge$5.5 classifiers are the lowest and highest potency BSEP models that we build. The active ratio in the training set, when potency threshold is at 4.5 and 5.5, is 0.825 and 0.400, respectively. We are investigating whether we can repurpose the pIC50$\ge$4.5 model to retrieve highly potent compounds at pIC50$\ge$5.5 (**Hypothesis 1**), and whether our method outperforms the high potency pIC50$\ge$5.5 model (**Hypothesis 2**).

**Hypothesis 1** holds, as shown in Table  3. Models $[20\; \upmu {\text{M}} / 0.16\; \upmu {\text{M}} / 4.5]$, $[20\; \upmu {\text{M}} / 0.8\; \upmu {\text{M}} / 4.5]$ and $[20\; \upmu {\text{M}} / 4\; \upmu {\text{M}} / 4.5]$ specifically retrieve compounds with pIC50$\ge$5.5, with significantly less false positives than the $[20\; \upmu {\text{M}} / 20\; \upmu {\text{M}} / 4.5]$ model.

In this case, the high potency model pIC50$\ge$5.5 is the best model in terms of high potency AUC-PR and AUC-ROC (Table  3), indicating that there are still enough positive samples for training of the high potency model. Our method does not lead to an improvement in high potency AUC-PR or AUC-ROC, hence **Hypothesis 2** does not hold.

### Immunology on-target assay

This is an assay for an immunology protein target. pIC50$\ge$5.3 and pIC50$\ge$6 classifiers are the lowest and highest potency models that we build for this assay. Hence, for this case we are investigating whether we can repurpose the pIC50$\ge$5.3 model to retrieve highly potent compounds at pIC50$\ge$6 ((**Hypothesis 1**)), and whether our method outperforms the high potency pIC50$\ge$6 model (**Hypothesis 2**). The active ratio in the training set, when potency threshold is at 5.3 and 6, is 0.437 and 0.0765.

In terms of **Hypothesis 1**, we observe a trend of increasing high potency precision as image concentration for inference decreases (Table  4), indicating that the model can be repurposed for highly potent compound retrieval. However, the high potency precision increase for this assay is smaller than in other cases, from 0.739 at $20\; \upmu {\text{M}}$ to 0.757 at $0.8\; \upmu {\text{M}}$, and the increase is not as monotonic.

Despite the low active ratio 0.0765, the high potency model $[20\; \upmu {\text{M}} / 20\; \upmu {\text{M}} / 6]$ performs well with AUC-ROC score of 0.718 and AUC-PR score of 0.258. But our method, specifically models $[20\; \upmu {\text{M}} / 0.8\; \upmu {\text{M}} / 5.3]$ and $[20\; \upmu {\text{M}} / 4\; \upmu {\text{M}} / 5.3]$, improve on these scores, achieving 0.347 AUC-PR and 0.821 AUC-ROC, and 0.328 AUC-PR and 0.861 AUC-ROC, respectively. This indicates **Hypothesis 2** holds for this assay.

**Table 4:** Immunology target metrics table. A high potency precision increase as inference image concentration decreases can be observed, indicating that the pIC50$\ge$5.3 model has been repurposed for retrieving highly potent compounds with pIC50$\ge$6. However, the increase is smaller and not as monotonic as the previous cases. The best model in terms of high potency AUC-ROC is $[20\; \upmu {\text{M}} / 4\; \upmu {\text{M}} / 5.3]$, and in terms of high potency AUC-PR is $[20\; \upmu {\text{M}} / 0.8\; \upmu {\text{M}} / 5.3]$, both outperforming the conventional method $[20\; \upmu {\text{M}} / 20\; \upmu {\text{M}} / 6]$.

Model name	High potency precision	High potency AUC-PR	High potency AUC-ROC
$[20\; \upmu {\text{M}} / 0.16\; \upmu {\text{M}} / 5.3]$	0.667 (10/15)	0.183	0.652
$[20\; \upmu {\text{M}} / 0.8\; \upmu {\text{M}} / 5.3]$	**0.757 (28/37)**	**0.347**	0.821
$[20\; \upmu {\text{M}} / 4\; \upmu {\text{M}} / 5.3]$	0.709 (39/55)	0.328	**0.861**
$[20\; \upmu {\text{M}} / 10\; \upmu {\text{M}} / 5.3]$	0.698 (37/53)	0.313	0.846
$[20\; \upmu {\text{M}} / 20\; \upmu {\text{M}} / 5.3]$	0.739 (34/46)	0.278	0.804
$[20\; \upmu {\text{M}} / 20\; \upmu {\text{M}} / 6]$	0.727 (16/22)	0.258	0.718

Highest values are in bold.

### Glu/Gal assay

Another important off-target assay in early drug development is Glu/Gal. Glu/Gal is the primary assay of choice for drug-induced mitochondrial toxicity^16^. Briefly, mitochondrial dysfunction is determined by the ratio of the test substance to induce cytotoxicity in glucose and galactose culture conditions^1^, hence the Glu/Gal nomenclature. The measure of mitochondrial toxicity is a ratio of two IC50 values, not one pIC50 value as in previous cases. The higher the Glu/Gal ratio is, the more indicative that the compound induces mitochondrial toxicity.

Instead of retrieving highly potent compounds, in this case we will test whether our method can specifically retrieve highly toxic compounds from this assay. We consider compounds with Glu/Gal ratio $\ge$5 to be highly toxic, and ratio $\ge$2 to be moderately toxic. The active ratio in the training set, when toxicity threshold is at 2 and 5, is 0.595 and 0.275, respectively. It is also worth noting that the Glu/Gal ratios are distributed on a wide range (up to 500), but we are only interested in the thresholds in the narrow range of 2 to 5. This is because we consider every compound with ratio $\ge$5 to be equally toxic. As a result, compounds in the test set will only have Glu/Gal ratios between 0 to 20.

It can be observed in Table  5 that high toxicity precision increases from 0.483 of model $[20\; \upmu {\text{M}} / 20\; \upmu {\text{M}} / 2]$ to 1 of model $[20\; \upmu {\text{M}} / 0.8\; \upmu {\text{M}} / 2]$. This shows using low concentration images for inference can specifically retrieve highly toxic compounds. This shows that **Hypothesis 1** holds for this assay. We note that similar to PLD, in this assay inference using $0.16\; \upmu {\text{M}}$ images returns no active compounds. It is because the compound concentration is too low that no signal related to these activities are induced in the cell.

Regarding **Hypothesis 2**, the high toxicity model $[20\; \upmu {\text{M}} / 20\; \upmu {\text{M}} / 5]$ remains the best performing model (Table  5) at 0.226 high toxicity AUC-PR and 0.713 high toxicity AUC-ROC. In this case, 27$\%$ of the labels are positive for classification of highly toxic compounds with Glu/Gal ratio$\ge$5, which is plenty of positive examples for a high toxicity model training. Our method does not outperform the connventional method for this assay.

**Table 5:** Glu/Gal metrics table. High potency precision tends to increase as inference image concentration decreases. This indicates the Toxicity$\ge$2 model has been repurposed for retrieving highly potent compounds with Toxicity$\ge$5. The best model in terms of high potency AUC-ROC and AUC-PR is $[20\; \upmu {\text{M}} / 20\; \upmu {\text{M}} / 5]$. In this case, our method does not outperform the conventional method.

Model name	High toxicity precision	High toxicity AUC-PR	High toxicity AUC-ROC
$[20\; \upmu {\text{M}} / 0.16\; \upmu {\text{M}} / 2]$	0 (0/0)	0.0	0.5
$[20\; \upmu {\text{M}} / 0.8\; \upmu {\text{M}} / 2]$	**1 (2/2)**	0.0155	0.508
$[20\; \upmu {\text{M}} / 4\; \upmu {\text{M}} / 2]$	0.654 (17/26)	0.0679	0.552
$[20\; \upmu {\text{M}} / 10\; \upmu {\text{M}} / 2]$	0.553 (57/103)	0.165	0.650
$[20\; \upmu {\text{M}} / 20\; \upmu {\text{M}} / 2]$	0.483 (87/180)	0.186	0.693
$[20\; \upmu {\text{M}} / 20\; \upmu {\text{M}} / 5]$	0.531 (85/160)	**0.226**	**0.713**

Highest values are in bold.

### Discussion

We have presented an approach, improving on an existing image-based small molecule activity optimization pipeline, to specifically retrieve highly potent compounds in a biological assay. We start with training a moderate-potency model with $20\; \upmu {\text{M}}$ cell painting images to classify compounds with pIC50 at a potency threshold low enough so that there are still plenty of positive examples to train effectively. Then, we repurpose that well-performing model for higher potency classification, by performing inference using lower concentration images as input. In terms of application in the drug discovery pipeline, being able to classify highly potent compounds accurately can help prioritizing hits from screening for experimental follow-up based on potency. It can also help deprioritizing compounds with potent off-target activities in the hit-triaging phase. However, it should be mentioned that the improvement in retrieval of highly potent compounds with this approach comes at a cost for data generation since to benefit from our approach, additional cell painting images of different concentrations are required.

We highlighted two points that our approach can achieve. Hypothesis 1 is that a good moderate-potency model can be repurposed to specifically retrieve compounds with higher potency, by using features from low concentration images for inference without retraining the model. We assess this point by using precision when classifying a highly potent compound, on 57 assays and 4 additional assays in the Case Studies section. We found that this behavior can be observed in almost all assays we tested on, with the majority of them being cell proliferation assays. Although using low concentration images for inference retrieves fewer active compounds, these compounds tend to be highly potent.

Hypothesis 2 was that if data imbalance adversely affected a high potency model training, our approach could outperform the high potency model (conventional method) in classifying highly potent compounds. We assessed this point on the same selection of assays as above, using AUC-ROC and corrected AUC-PR as metrics. We found that this point holds for around 65$\%$ to $75\%$ of those assays. Overall, AUC-ROC scores increase by around 0.1 to 0.2, and AUC-PR scores increase by around 0.2 to 0.5, indicating an improvement over the conventional method in the majority of assays. Our approach can serve as a replacement for a conventional high potency model when activity labels for training are scarce.

## Supplementary Information

Supplementary Information.

## Data Availability

The data that support the findings of this study (Cell Painting and molecule activity data) are proprietary of Johnson & Johnson, and, therefore, cannot be made available. For more information about the data, please contact Steffen Jaensch at sjaensch@its.jnj.com.^12^
