# Supplementary material for: Low concentration cell painting images enable the identification of highly potent compounds
Source: Sci Rep. 2024 Oct 17;14:24403. doi: 10.1038/s41598-024-75401-5 (PMC11487191; doi:10.1038/s41598-024-75401-5)
Supplement: Supplementary file 1 — Supplementary Information. [file 41598_2024_75401_MOESM1_ESM.pdf]

Supplementary Information

August 22, 2024

1 Model Architecture and Additional Details about Training Procedure

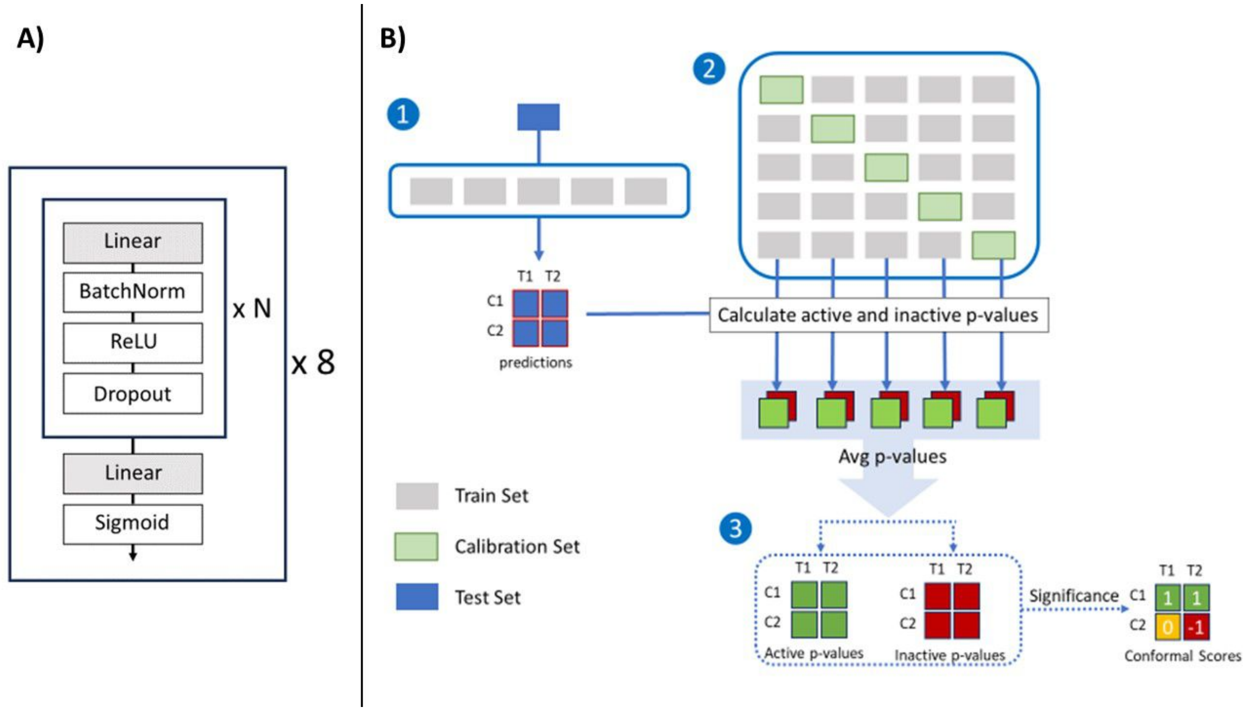

Figure 1: **A) Architecture of the model.** The model is an ensemble of 8 Multi-Layer Perceptrons, with N 'blocks' consisting of a Linear layer, BatchNorm, ReLU activation and Dropout. At the end of the model is the final linear layer (corresponding to the end tasks) followed by sigmoid to produce the probability scores for the different end tasks. **B) Training and Inference Process.** Firstly, the model is trained on the entire train set, and inference is done on the test set (for example, compounds C1, C2 in tasks T1, T2). Then, MCCP is performed for the 5 folds of the training set, acquiring an active p-value and an inactive p-value for each output probability score. Finally, the conformal scores for the outputs are calculated using the p-values, which returns 1 for Active, -1 for Inactive, and 0 for Uncertain.

Model Hyperparameters

Model	Hidden Layers Sizes	Dropout	Learning Rate	Weight Decay
0	(512, 512, 512, 512)	0.5	0.0005	0.0
1	(1536, 1536, 1536)	0.3	0.0001	0.0
2	(256, 256)	0.5	0.0001	1e-05
3	(1024, 1024)	0.1	0.0005	0.0001
4	(1024, 1024, 1024)	0.5	0.0001	0.0001
5	(2048, 2048, 2048)	0.3	5e-05	1e-05
6	(512, 512, 512)	0.0	5e-05	1e-05
7	(1024, 1024)	0.1	1e-05	1e-05

Table 1: **Hyperparameters for each of the MLP in the ensemble.**

2 Stem Plots Visualization of Assays in Case Study

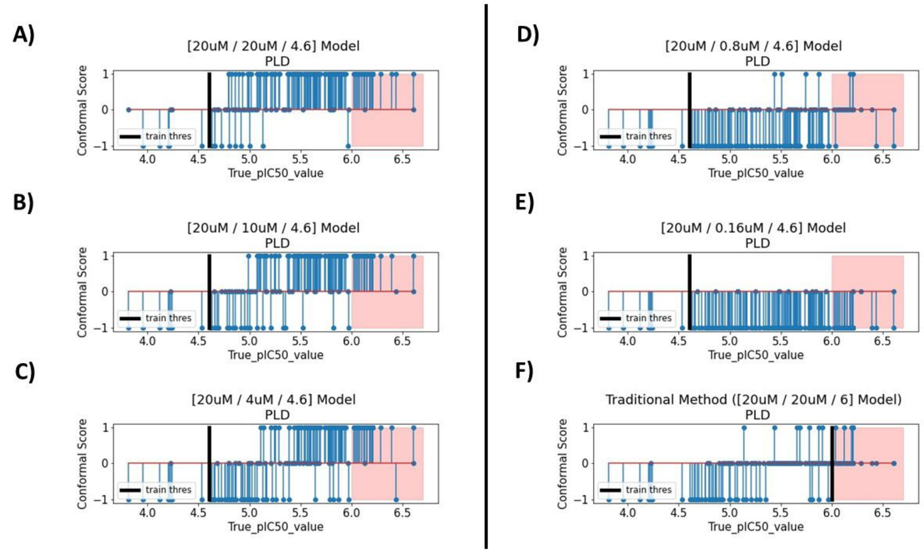

Figure 2: **PLD Assay**

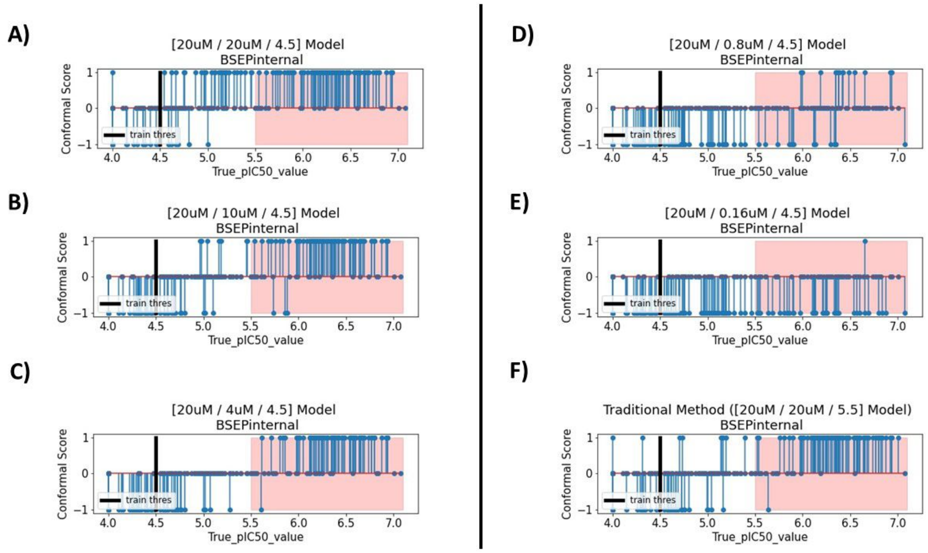

Figure 3: **BSEP Assay**

3 Cell Microscopy Images Sample

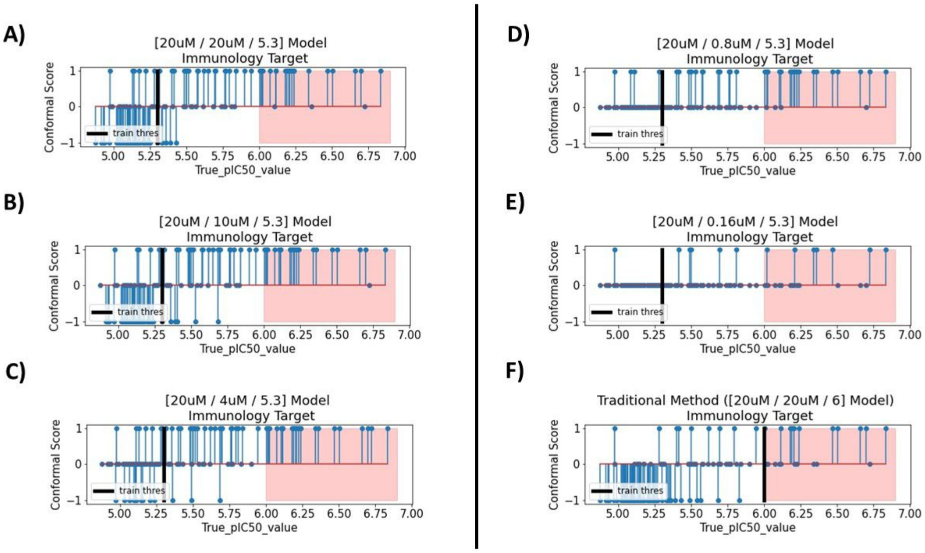

Figure 4: **Immunology Target Assay**

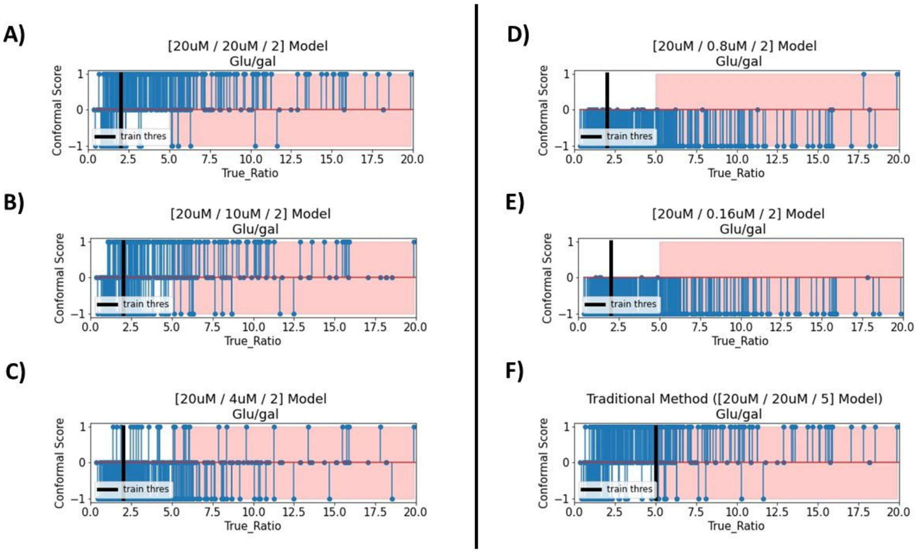

Figure 5: **Glu/gal Assay**

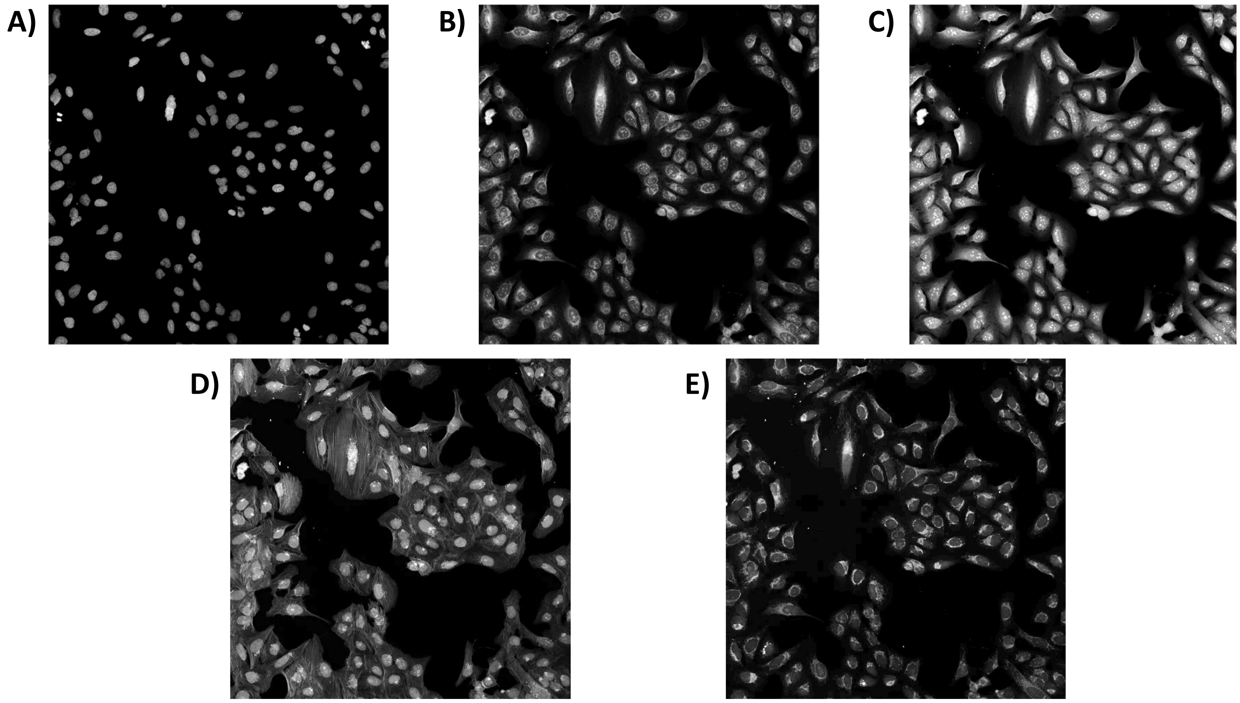

Figure 6: **Sample cell painting microscopy images.** Five channels of the same view imaged in the Cell Painting protocol. Each highlights a different organelle or cellular component, A) Nucleus, B) Endoplasmic reticulum, C) Nucleoli, cytoplasmic RNA, D) Actin, Golgi, plasma membrane, E) Mitochondria.
